# “I would never have become an interpreter without social media”: online social media’s motivational dynamics in self-regulated interpreting learning

**DOI:** 10.3389/fpsyg.2025.1499584

**Published:** 2025-06-20

**Authors:** Zhiyu Cai, Shanshan Yang

**Affiliations:** ^1^Graduate School of Translation and Interpreting, Beijing Foreign Studies University, Beijing, China; ^2^Department of English, School of Foreign Languages, Central China Normal University, Wuhan, Hubei, China

**Keywords:** motivation, motivation regulation, self-regulated learning, online social media, interpreting learners

## Abstract

**Introduction:**

In the Web 2.0 era, self-regulated learning (SRL) through online social media (OSM) has become integral to language learning, particularly for Chinese interpreting learners preparing for postgraduate programs. Despite its growing role, the impact of OSM on interpreting learners’ motivation and SRL outcomes remains underexplored.

**Methods:**

Grounded in Zimmerman’s Cyclical Model of SRL, this study employed semi-structured interviews with 29 Chinese interpreting learners to examine OSM-mediated motivational dynamics throughout the Self-regulated interpreting learning process.

**Results:**

Our findings reveal that OSM acts as a double-edged sword. OSM significantly enhances learners’ motivation, operating both as an end-state and an ongoing process, by providing accessible resources, offering emotional support, creating platforms for community engagement, increasing interpreter visibility, and offering learning affordances that translate initial motivations into actions. Meanwhile, OSM also acts as a deterrent to and regulator of motivation due to its entertaining and social nature, which can introduce distractions, misinformation, and increased anxiety among interpreting learners, potentially hindering the effective regulation of motivation.

**Discussion:**

Our findings suggest that navigating OSM effectively requires the development of critical thinking skills. This research contributes to the understanding of OSM-mediated interpreting education and offers insights into how educators can help learners harness the benefits of OSM while mitigating its drawbacks.

## 1 Introduction

Bilibili^1^, a widely-used Chinese Online Social Media (OSM) platform, is often humorously referred to as a “learning platform,” which underscores the growing educational role of OSM in contemporary society. In the Web 2.0 era, OSM have become an indispensable tool in higher education (Dabbagh and Kitsantas, 2012; Gikas and Grant, 2013; Manca, 2020), significantly transforming the landscape of language learning (Zainuddin and Yunus, 2022; Zhao and Yang, 2023; Meirbekov et al., 2023; Jin, 2023). This trend is evident in interpreting, a skill involving real-time language mediation, where an expanding community of learners engages in various forms of online learning. In China particularly, this phenomenon has gained significant momentum, with increasing numbers of interpreting enthusiasts proactively turning to online resources and platforms, some of whom have not necessarily been exposed to formal interpreting education, particularly students preparing for admission to Master’s program in interpreting. In this context, self-regulated learning (SRL) has emerged as a predominant method of learning.

Within the framework of “geopolitics of pedagogy” (Cronin, 2005, p. 254), it is vital that pedagogical approaches address local conditions (Laviosa and Falco, 2022). However, the uneven distribution of interpreting education resources is a global issue, and China is no exception. While the European Union and North America offer well-developed interpreting training systems (Amato and Mack, 2022), many regions in the Global South, including parts of Africa, Asia, and Latin America, face significant challenges in accessing quality interpreter training (Afolabi and Oyetoyan, 2022). In China, although major cities like Beijing and Shanghai host well-established training programs at renowned institutions, more peripheral regions face limited or subpar resources. Despite these disparities, OSM has emerged as a valuable alternative, making interpreting education more accessible globally. This trend, though particularly prominent in China, mirrors a broader global shift toward informal, online learning platforms that increasingly supplement formal educational structures.

Online social media offers a unique blend of knowledge, social interaction, and entertainment, creating an authentic learning environment (González-Davies and Enríquez-Raído, 2016) that potentially reshapes the “being and becoming” of interpreting learners, influencing their motivation, ultimately their competence and performance. Despite its growing role in learning, research specifically addressing the influence of OSM on interpreting learners’ motivation, particularly in the context of SRL, remains limited. Motivation is a key driver of SRL, impacting both the initiation and regulation of learning efforts.

Motivation in SRL can be seen as both a product and a process (Winne and Marx, 1989; Wolters, 2003), with the latter emphasizing the regulation of motivation. While Wolters (2003) emphasizes the importance of motivational regulation within SRL, much of the existing literature on interpreting has treated motivation as a static end-state, often examining it in relation to learners’ aptitude, career orientation, or pedagogical application (Wu, 2016). This approach overlooks the dynamic nature of motivation and its regulatory processes. To date, although there has been some recognition of OSM’s role in interpreter training (González-Davies and Enríquez-Raído, 2016; Chang and Wu, 2017; Valtchuk and Class, 2021), comprehensive studies on its impact remain scarce.

To better understand how motivation functions within the context of Self-Regulated Interpreting Learning (SRIL), it is crucial to explore the dynamic relationship between OSM and motivation, particularly its regulatory role in the SRIL process. Building on existing research into interpreting learners’ motivation and SRL (Lung, 2005; Timarová and Ungoed-Thomas, 2008; Yan et al., 2010; Pöchhacker and Liu, 2014; Melicherčíková and Dove, 2021; Liu, 2022; Liu and Zhang, 2022), this study seeks to address this gap by examining the motivational dynamics of OSM in SRIL through the lens of Zimmerman’s Cyclical Model of SRL. Using semi-structured interviews with 29 Chinese interpreting learners, the study offers new insights into how OSM shapes learners’ motivation during SRIL. By focusing on the experiences of interpreting students in China, this research contributes to a developing field that requires further exploration.

## 2 Literature review

### 2.1 Motivation and interpreting learning

Motivation is a key psychological determinant influencing the outcome of second/foreign language acquisition (Dörnyei, 1990). As a more complex and cognitively demanding form of language use, interpreting requires sustained and intensive practice, evidently more sensitive to motivation. Research on motivation in interpreter training predominantly adopts a function-oriented approach and mainly falls into three categories: learners’ aptitude (Timarová and Ungoed-Thomas, 2008; Pöchhacker and Liu, 2014; Liu and Zhang, 2022), career orientation (Lung, 2005; Yan et al., 2010) and pedagogical utilization (Melicherčíková and Dove, 2021; Liu, 2022).

Motivation, commonly regarded as a “soft skill” in aptitude tests, is seen as a variable predicative of individual differences to determine whether a student is more teachable and more likely to succeed. Highly motivated students, particularly those engaged in SRL, are more likely to succeed in interpreting courses (Melicherčíková and Dove, 2021), exhibit greater cognitive flexibility, and are less vulnerable to anxiety (Timarová and Salaets, 2011), while others refute the notion that strong motivation predicts or guarantees good performance (Liu, 2022; Liu and Zhang, 2022). Moreover, motivation is viewed as a by-product of individuals’ career orientation, serving as a “social ladder” (Lung, 2005, p. 230) and for job seeking (Yan et al., 2010). Nevertheless, empirical research on external or sociological factors influencing learners’ motivation remains scant.

Pedagogically speaking, current studies on interpreter trainees’ motivation have touched upon a range of (de)motivating factors. For instance, intrinsic motivation, ideal self, mastery and significant others are found to be among the strongest motivators, yet altruism appears to be a drawback (Horváth and Kálmán, 2020). Factors related to self, peer, teacher, and institute-attributed also possibly erode trainees’ motivation (Wu, 2016). High-stakes exams and practice can either make or break students’ motivation (Liu, 2022). Several interpreter learning motivation scales (Cai and Dong, 2017) have been developed in an effort to conceptualize motivation in interpreting learning. However, the evolving trends of new factors, particularly those brought by online learning communities on OSM, and their impact on the motivation of interpreting learners have not been adequately addressed in current literature.

To conclude, from trainers’ perspective, trainees’ motivation is often valued in relation to measurable learning outcomes as an end-state product rather than the catalyzer of an ongoing process. This perspective overlooks the dynamics that emerge from the actual learning process, which largely relies on students’ self-regulatory skills, and how motivational belief affects trainee interpreters’ application of SRL strategies.

### 2.2 Interpreting and SRL

**FIGURE 1:**
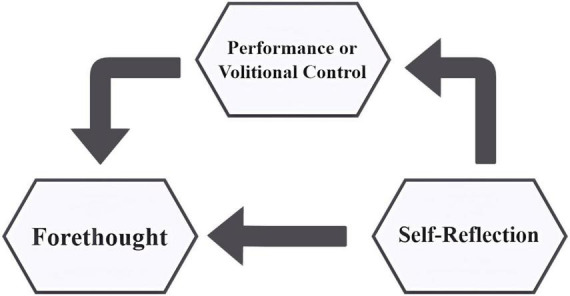
Cyclical phases model (1st version). Adapted from Zimmerman (2000).

Self-regulated learning encompasses cognitive, metacognitive, behavioral, motivational, and emotional/affective aspects of learning (Panadero, 2017). Zimmerman (2000, p.14) defines it as “self-generated thoughts, feelings, and actions that are planned and cyclically adapted to the attainment of personal goals.” Cyclical Phases model (see Figure 1) offers a robust framework for understanding how motivation and self-regulation interact during the learning process. In this model, self-regulatory processes and beliefs are divided into three cyclical phases: forethought, performance, and self-reflection. Forethought precedes actions and sets the stage for them. Performance, or volitional control, occurs during efforts, affecting attention and action. Finally, self-reflection takes place after efforts, and influences a person’s response to the experience. These reflections, in turn, inform the forethought phase for subsequent efforts, completing the cycle of self-regulation (Zimmerman, 2000).

**FIGURE 2:**
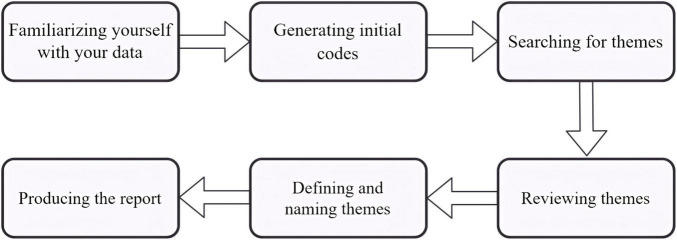
Phase structure and sub-processes of cyclical self-regulation.

As shown in Figure 2, motivational beliefs, as end-state products, play a significant role, while motivation as a process is also relevant to its regulation (Wolters, 2003). Motivational regulation, as an integral component of SRL, is typically defined as thoughts and actions through which students deliberately try to influence their own motivation or motivational processing to achieve optimal academic outcomes (Brown et al., 1989; Zimmerman and Schunk, 2008). Recent trends in SRL motivation predominantly focus on new personal learning environments (Xu et al., 2024), such as online learning (Kara et al., 2024), virtual reality (Wang et al., 2024), mobile-assisted learning (Ma and Chiu, 2024), game-based learning (Lin et al., 2024; Zhang et al., 2024) and Generative AI (Chiu, 2024).

The nature of interpreting learning endows a pivotal role to SRL in both traditional and innovative interpreting pedagogy. As part of this process, students are often tasked or encouraged to engage in solo practice, group practice and peer evaluation. Current research on SRIL can be broadly classified into two main categories. The first examines SRIL itself from perspectives such as learner factors, learners’ subjective initiative, and learning outcomes. Case studies exploring interpreter training programs across countries reveal the pivotal influence of SRIL, highlighting its crucial role in shaping future interpreters (Hansen and Shlesinger, 2007; Horváth, 2007). Additionally, more nuanced aspects, including learner factors such as gender, motivation, personal habits (Yan et al., 2010), and personality (Hodáková, 2021), are found closely related to the SRIL process. Learners’ subjective initiatives, such as self-monitoring capability (Bakti and Bóna, 2016), self-perceived language ability (Yan et al., 2010), self-assessment and learners’ diaries (Mraček and Vavroušová, 2021), are also significantly relevant. Generally, the SRIL approach reports positive outcomes, such as increased efficiency, independent thinking ability, and reduced anxiety (Hansen and Shlesinger, 2007). However, the recent dynamics of current SRL studies, especially students’ online engagement, haven’t yet been included in interpreting pedagogy research.

Meanwhile, the facilitation and application of SRIL has been extensively explored, evidenced by scholarly efforts to develop online self-learning platforms (Hansen and Shlesinger, 2007; Hui, 2019). Integrating e-learning platforms to facilitate student SRIL is highlighted, particularly in providing access to authentic speeches and live-streaming of conferences as learning materials (Hansen and Shlesinger, 2007). While several e-learning platforms, such as Speech Repository^2^, offer resources for interpreting learners, scholars have explored the connection between these resources and SRIL (Hui, 2019). Beyond that, initiatives facilitated by information and communication technologies, such as the Computer-Assisted Interpreter Training system (Amato and Mack, 2022), combined with the trend of blended teaching methods and distance interpreter education due to the COVID-19 pandemic (Ren, 2020; Zhang and Ding, 2021), have together fostered greater engagement in SRIL among students.

Given its factual importance, SRIL has become more applicable for teachers and more accessible for students. However, a significant gap for SRIL is the restricted access to online platforms created by educational institutions, which can only be accessed by their own students on campus (Hui, 2019). Consequently, learners are compelled to explore alternative avenues, such as relying on resources available on OSM platforms. This trend is becoming increasingly evident, yet existing research has paid little attention to SRIL on OSM platforms.

### 2.3 OSM and language/interpreting learning motivation

The potential effectiveness of OSM-driven pedagogy has garnered significant attention among educational scholars. Extensive research has explored the impact of OSM on students’ motivation and academic performance. For instance, online distant learning tools, like MOOCs and OSM are argued capable of enhancing students’ intrinsic motivation (Kaplan and Haenlein, 2016); Alt (2015) found that engagement with social media can predict college students’ academic motivation, indicating a positive correlation between social media use and academic drive. More specifically, Erhel et al. (2022) demonstrated that a Twitter-based self-learning approach not only increased students’ intrinsic motivation and interest but also reduced amotivation.

Studies focusing on language learning report similar findings regarding the motivational potential of OSM. Zainuddin and Yunus (2022) reviewed that social networking sites serve as a bridge between formal and informal English language learning, leading to observed improvements in both learners’ enjoyment and motivation. From the perspective of educators, various studies have demonstrated the benefits of incorporating social media into language learning. For example, in Teaching English as a Foreign Language (TEFL) settings, social media-supported flipped classroom can improve learners’ writing performance by reducing anxiety (Zhao and Yang, 2023); Using Instagram and Tik Tok as video teaching tools in EFL classes can boost students’ motivation and interest (Meirbekov et al., 2023). Additionally, media-integrated activities, like vlogging, can also improve EFL learners’ motivation and speaking proficiency (Jin, 2023). However, these studies are limited to the classroom setting, where social media serves as a pedagogical supplement. There is insufficient research on students’ self-initiated practice on OSM.

Initial discussions on the role of OSM in interpreting learning motivation are closely tied to the concepts of Situated Learning and Community of Practice. González-Davies and Enríquez-Raído (2016) emphasized that global social media practices play a crucial role in situated learning in interpreting by creating an authentic learning environment, enabling interpreting students to engage in real-world contexts and practices. Aligning with this perspective, Chang and Wu (2017) suggest that interpreting students are encouraged to join online interpreter communities by subscribing to interpreters’ blogs and befriending interpreters on Facebook. Such exposure to authentic settings and connections with professional interpreters can significantly benefit student interpreters’ perception and motivation. In a preliminary empirical investigation on OSM-driven interpreting pedagogy, Valtchuk and Class (2021) focus on non-institutional group chats and situated learning on Facebook, shedding light on how social networking tools can be used beneficially in interpreting learning. However, there is a notable gap in understanding how students fulfill this task and the specific role OSM plays in this process, especially regarding their motivational regulation.

### 2.4 Rationale and research questions

A remarkable hiatus is identified in the scholarly investigation of motivation among self-regulated learners within the context of interpreting, with scant attention paid to the implications of OSM on this aspect. Existing studies predominantly adopt a trainer’s perspective rather than a student-initiated approach, emphasizing institutionalized practices over spontaneous, authentic learning processes. Consequently, motivation has largely been examined and discussed in classroom settings, thus overlooking students’ subjectivity and its interaction with practical factors such as industry dynamics. Viewed through a sociological lens, OSM functions as a unique actant that bridges individual thoughts and external forces. In this context, interpreting learners’ motivation should be perceived as an ongoing process rather than a static product. In line with the sequential progression inherent in interpreting learning, exploring learners’ evolving motivation requires a phase-by-phase approach, elucidating the interaction of each phase with OSM.

To address this lacuna, this study tries to investigate the evolution of learners’ motivations in SRL setting from the perspective of OSM users. We draw on Zimmerman’s (2000) three-phase Cyclical model to answer the following research questions:

1 How does OSM (de)motivate interpreting learners in SRL?
2 How does OSM help interpreting learners translate their motivations into actions?
3 How does OSM influence interpreting learners in regulating their motivations during SRL?

## 3 Materials and methods

### 3.1 Research setting and participants

This study interviewed Chinese interpreting learners about their motivation of self-learning interpreting using Chinese domestic OSM platforms including Sina Microblog^3^ (Xinlang Weibo), Little Red Book (Xiaohongshu^4^), Bilibili, and WeChat^5^ Official Account (Weixin Gongzhonghao). These platforms were chosen not only for their popularity among Chinese youth, but also for their multimodal capabilities of sharing texts, pictures, audio, and videos, creating diverse learning environments for interpreting practice.

Semi-structured interviews were chosen to broadly capture interpreting learners’ perceptions and experiences regarding how their motivations are engaged in OSM. The sampling strategy targeted Chinese interpreting learners actively engaged with OSM, with inclusion criteria emphasizing (1) OSM usage for interpreting learning at “Random,” “Regular” and “Frequent” levels, (2) foundational interpreting skills and basic knowledge, at least beginner-level consecutive interpreting proficiency, and (3) experience with authoritative interpreting tests in China such as China Accreditation Test for Translators and Interpreters (CATTI), or Professional Graduate Examination in English (PGEE), or real-world interpreting practice.

To ensure theoretical saturation (Glaser and Strauss, 2017), a total of 29 interpreting learners who met these requirements were engaged. The sample spanned various demographics, including individuals across genders (24 females, and 5 males), ages (20–29, *M* = 23.75, SD = 1.79), and learning duration (0.5–6 years, *M* = 3.27, SD = 1.33). Participants included PGEE exam candidates (*n* = 1), MTI and MA graduates (*n* = 26) and professionals in the workforce (*n* = 2). Academic backgrounds varied between English majors (*n* = 14) and non-English majors (*n* = 15), with participants from China’s top-ranked foreign language universities (*n* = 12), comprehensive key universities without foreign language specialization (*n* = 15), and non-key public universities or private colleges (*n* = 2). Among the participants, 19 had random real-life interpreting experience (liaison or consecutive interpreting). This diverse sampling aimed to mitigate bias and deepen understanding of SRIL across varied educational contexts.

Participants were recruited via social networks (*n* = 13), social media platforms (*n* = 5), and personal contacts (*n* = 11). During interviews, participants reflect on their motivation of interpreting learning, self-learning process on OSM, preparation for different interpreting exams, and personal insights into motivation regulation. In sum, all participants demonstrated sufficient motivation to engage meaningfully in the interview process.

### 3.2 Data collection and procedures

Semi-structured interviews were conducted from January 2023 to February 2024 via Tencent Meeting or in-person. The interview guides were produced in Chinese and translated into English by the authors (see Appendix 1 for the interview protocol). Given the exploratory nature of the study, questions were deliberately crafted to be open-ended, allowing emerging discussions to shape the direction of the interviews.

Mandarin Chinese was chosen as the interview language to prioritize participants’ native language expression. A total of 1134 min of interview data was recorded (averaging 46 min per interview). Audio recordings were transcribed using iflyrec (Xunfeitingjian), a speech-to-text transcription software developed by iFLYTEK., and subsequently imported into NVivo11 for data analysis. It should be noted that the quotes presented in the section “4 Findings” were translated from Chinese to English by the authors under the principle of preserving the original meaning while ensuring clarity for an English-speaking audience.

Ethical considerations were carefully addressed throughout the research process. All participants voluntarily joined the study after being fully informed of its purpose and nature, and signed consent forms before participating. Pseudonyms were assigned to ensure participant anonymity, and participants were informed of their right to withdraw at any point.

### 3.3 Data analysis

**FIGURE 3:**
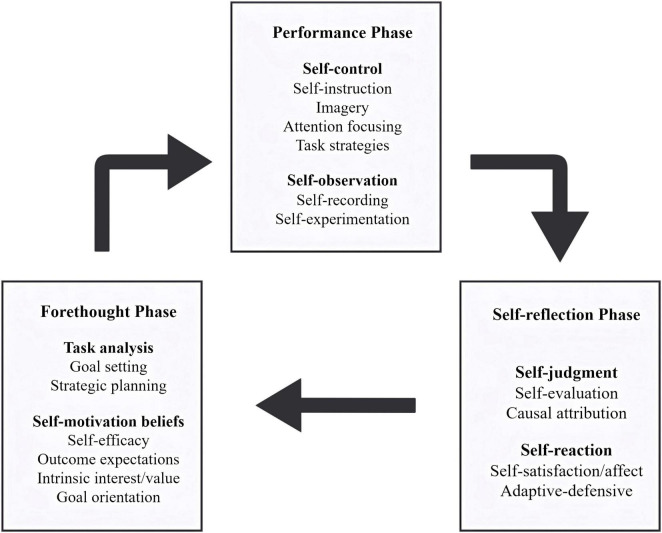
Systematic process for thematic data analysis. Adapted from Nowell et al. (2017).

We employed thematic data analysis following the systematic process described by Nowell et al. (2017), which involves six steps (see Figure 3): familiarizing yourself with your data, generating initial codes, searching for themes, reviewing themes, defining and naming themes, and producing the report to enhance the reliability of qualitative analysis.

Our analytical strategy combined deductive and inductive approaches. The main categories were derived from Zimmerman’s (2000) three phases of SRL: forethought, performance, and self-reflection (top-down/deductive). Within these established categories, sub-categories were developed inductively from the data itself (bottom-up/inductive).

The coding process began with the first author thoroughly reading each transcript and initially coding relevant statements (see Appendix 2). With our research questions focusing on “motivation” and “motivational regulation,” our secondary coding directly corresponded to these central concepts. This was followed by a rigorous iterative process of reading, comparing, and discussing the data to identify key patterns and concepts. A representative coding example of the sub-categories identified through thematic analysis, corresponding to the main theme of forethought, is provided in Appendix 3.

This approach culminated in seventeen definitive sub-categories of codes presented in Table 1 in the section “4 Findings.” The relationships among these categories are presented as a diachronic narrative, demonstrating their evolution over time.

**TABLE 1:** A summary of categories and sub-categories for motivation in self-regulated interpreting learning (SRIL).

SRL phase	Status of motivation	Sub-category	Definition of sub-category related to SRIL	*N* = 29
**Forethought**	Formation	Interpreters’ image	Influenced by interpreter’s image created by OSM	5
		Subpar in-class teaching	Offline teaching is found far from satisfaction	25
		Role model	Drawing inspiration from successful examples	20
	Regulation	Goal setting	Setting goals before starting learning	29
		“experience post” guidance	Planning according to “experience posts”	28
**Performance**	Formation	Entertaining nature	OSM’s entertaining nature reduces uneasiness	10
		Emotional support	Contents on OSM are emotionally supportive	15
	Regulation	Speech bank	OSM serving as an accessible speech bank	29
		Virtual trainer	Content producers serving as virtual trainers	20
		Paid tutorial	Spending money to boost motivation	7
		Sharing as learning	Sharing as a self-learning strategy	8
		Learning venue	A virtual venue of practicing and reflection	6
	Dissolution	Psychological drawbacks	OSM sometimes dampen learners’ morale	24
		Misinformation	Learners can be misled by OSM content	9
**Self-**r**eflection**	Regulation	Goal renewal	Learners renew their phased goal guided by OSM	12
		Second-hand experience	learners can absorb second hand experience	6
		Job assistant	OSM helps learners to find job or internship	4
	Dissolution	Feedback loop	Using OSM as a complementary tool for feedback	21
		Exit behavior	Stop learning interpreting on OSM	17

To ensure the reliability of our findings, we engaged in open discussions with participants about the identified patterns. The researchers’ three-in-one position as researcher, student interpreter, and insider within the interpreting online community uniquely facilitated a conceptual, empathetic, and interdisciplinary exploration (Savvides et al., 2014). This multifaceted perspective was instrumental in navigating the dynamics of online communities and interpreting learning.

## 4 Findings

Table 1 presents the descriptive statistics of the main themes identified at each of the three time points, organized under the categories of forethought, performance, and self-reflection. The analysis specifically focuses on motivation’s evolution through a dynamic perspective, including motivation itself as a product and the regulation of motivation as a process; the former is further divided into its formation and dissolution.

### 4.1 To start or not to start: motivation’s evolution in forethought

Within Zimmerman’s SRL model, self-motivation emerges as a crucial element. Yet, the genesis of motivation for interpreting learning prompts further inquiry. For the emerging cohort of interpreting learners as “digital natives,” OSM has transitioned to become their predominant information source, thereby playing an indispensable pseudo-environment in sculpting their motivation. Several participants recognized OSM as a significant influence in shaping their perception of interpreting as a profession, a pivotal external driver of their motivation. A representative remark was made by Roma, aged 24:

So, Bao Gaogao’s^6^ simultaneous interpreting vlog popped up suddenly. It was around my junior to senior year of college. I was really torn about what major to pick for my grad school entrance exams, and when I stumbled upon that vlog, I just thought: “wow it was literally cool.”

Today’s interpreters, as active influencers on OSM, predominantly operate as freelancers, who tend to secure more market-oriented assignments and higher income. The image of interpreters has thus been constructed as individuals who are independent, skilled, financially rewarded, possess flexible schedules, and enjoy high social status, which has become one of the key factors attracting some learners to enter the profession.

However, for aspiring learners, the primary challenge arises as in-class teaching of interpreting at the undergraduate level is far from satisfactory. Participants’ feedback corroborates this finding. Tim, a 24 years learner, said, “I did my undergrad in English at an agricultural university. To be honest, it was pretty crappy. I didn’t really learn much from classes.” In line with Tim’s comments, a mere 10% of learners believed that undergraduate interpreting courses bolstered their educational experience. Notably, textbooks were outdated and instructors often lacked practical experience, not even to mention providing current professional insights. Additionally, teaching methods were disconnected from real-world applications, hindering students from gaining authentic professional experiences. Consequently, motivated learners often turn to OSM to compensate for inadequate in-classroom learning.

After examining external factors, the ensuing step for novice learners is to set a concrete goal. Aspiring learners soon realize that the industry mandates specific prerequisites for entry, requiring verification of their capabilities and expertise. In China, a student interpreter’s proficiency is primarily assessed through professional or academic qualifications, such as CATTI, the only authoritative vocational certificate in China, or PGEE. Julian, a 26-year-old technical writer and former interpreter, told me, “Enrollment in an institutionalized program signifies the beginning of my interpreting journey.”

Once a goal is set, learners transit from novices to prospective candidates, requiring an operational plan to regulate their motivation. During the analytical process of coding and categorization, utilizing “experience posts” (经验贴, *Jingyantie*) emerged as a noteworthy theme of motivational regulation. This term encompasses a wide array of content, from exam preparation, time management strategies, and advice on program selection to practice techniques. Most posts are contributed by students who successfully passed relevant exams, with a minority from teachers within training institutions. For example, Miya, a 25-year-old interpreter graduate, firstly searched social media for relevant resources.

I chose a day to dig up all the posts I could find on social media, putting together everything they shared, like what the exam was like, what was on it, and even the sharers’ academic backgrounds. After I set a plan for Summer Camp^7^, I didn’t look up any more experience post.

**FIGURE 4:**
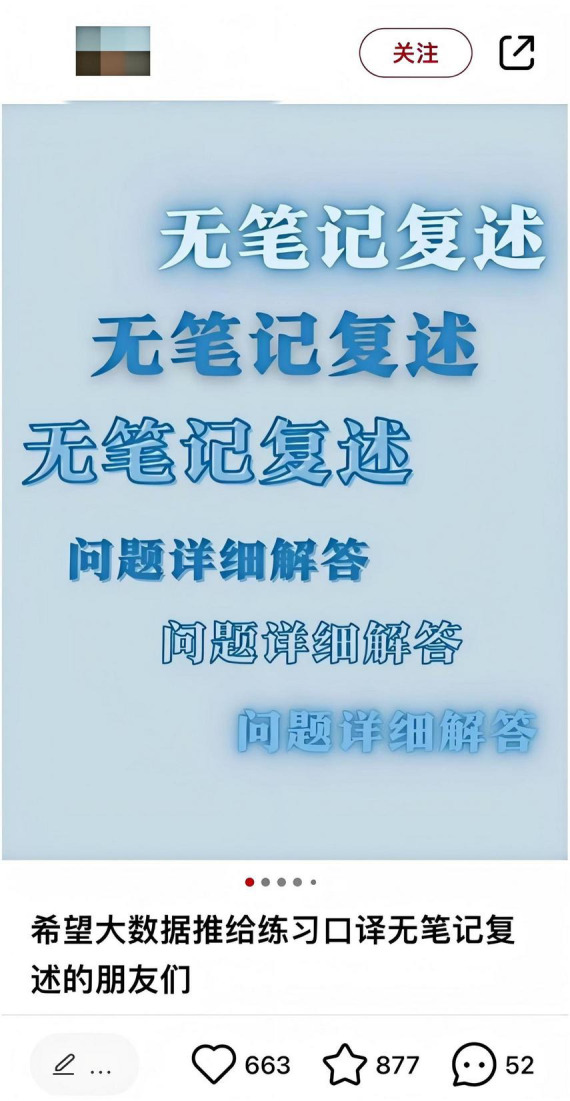
Cover page of Miya’s experience post on Xiaohongshu.

**FIGURE 5:**
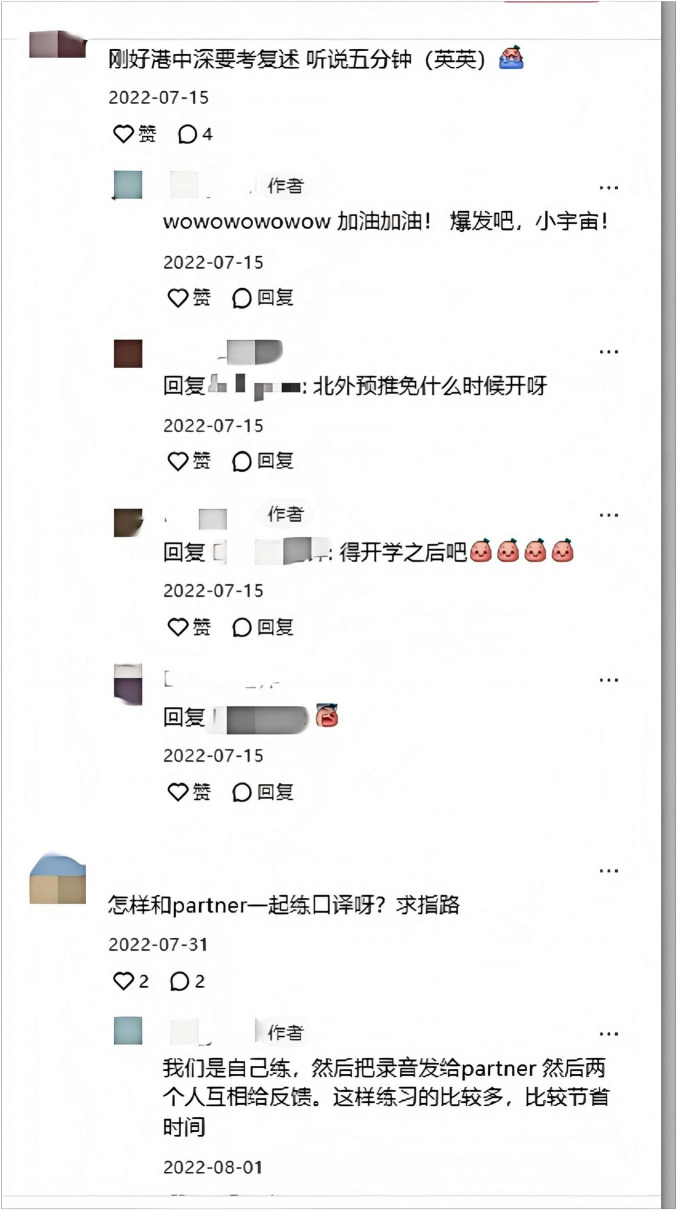
Comment section of Miya’s experience post.

Miya later became a contributor of interpreting-related knowledge on Xiaohongshu. Figure 4 presents the cover page of her experience post, where the large, bold font immediately draws attention. The purpose of Miya’s posts is to share her experience and encourage viewers to initiate and engage in interpreting practice. Figure 5 illustrates the real-time interaction in the comment section of her post. In her experience post and comments, an array of practical advice on partner-based interpreting, retelling strategies, and language foundation tips are shared.

Online social media also boosts morale by showcasing diverse role models. Although interpreting practice can be discouraging due to its inherent difficulty, the role models presented on OSM serve as benchmarks for predicting personal success, thereby making the pursuit of interpreting seem more attainable.

Chris, a 25-year-old interpreting student with an undergraduate background in Tourism Management, reflected on her change of mindset:

I used to lack confidence for being a non-English major and doubt if I could make it through the exam. But eventually, I passed, and so did many others in non-English majors. Maybe seeing me pass made others think, “if she can do it, maybe I can too.”

### 4.2 To continue or not to continue: diversified performances to regulate motivation

Once their practice begins, learners face two major challenges of motivational regulation: deciding what to practice and how to practice. The former pertains to the selection of practice materials, while the latter involves the choice of strategies and techniques. OSM has the potential to address both. “It is just like a grocery store. You have a vast array of knowledge and information at your hand” (Miya).

For newcomers, OSM primarily functions as a speech bank, or an alternative for institution-built e-learning platforms. OSM-based Speech Banks can be divided into several types. After being demotivated by inadequate offline teaching with unsuitable materials and methods, learners suddenly find easily accessible, relevant, up-to-date, and high-quality materials for practice. Additionally, they may find these resources easy to digest, with transcriptions, translations, note-taking demonstrations, explanations, and occasionally, interpreting recordings offered.

To address the question “how to practice,” OSM functions as a “virtual trainer” for novice learners. Neither the undergraduate education nor the graduate education covers every aspect. “Teachers assume I possess certain interpreting skills. Well, actually I do not,” said Susan, 24 years old. This brings more learners to OMS seeking tailored learning methods. The “grocery store” offers a wide selection of contents produced by a spectrum of contributors, including students, professionals, and training agencies. Consumers may include not only newcomers needing personalized solutions, but also English major students from prestigious language universities, among whom Joy is an example. She remembered:

Back when I was in my undergrad class, I couldn’t get how to quickly deal with numbers. After class, we’d dig around for stuff to practice with. My friend pointed me to this video on Bilibili. It was by this guy, also learning interpreting, who made a video sharing his own tricks. After watching that, we started using his method and we got the hang of interpreting numbers in no time.

Such “virtual trainers” on OSM covers an array of interpreting learning methods diversified in theme, structure, and formality. These range from conversational to simultaneous interpreting, spanning from comprehensive and systematic course series to individual and standalone entries, and vary from formal textbook methodologies to highly personalized practice techniques. These methods include techniques for formal interpreting activities, like note-taking, number interpreting, and the principle of syntactic linearity in simultaneous interpreting—and auxiliary skills for interpreting practice, such as shadowing, retelling, public speaking, and sight translation. Additionally, coping tactics for enhancing interpreting performance are also incorporated.

Despite materials and methods, 37.9% (*n* = 11) of learners also develop a unique way to regulate their motivation: paying for hand-hold teaching. These paid tutorials typically have a clear focus on PGEE or CATTI. Thea, a 24-years-old MTI student who had benefited from such classes, said:

If one is trained in public speaking, then he or she will naturally be a better public speaker. If another candidate, to say, shares the same language ability as mine, but has not been fully prepared by taking a similar tutorial for oral test, I think it would be quite a loss. Hence payment is acceptable.

According to Thea, despite the effectiveness of paid tutorials, spending money is also a self-regulated strategy that can make her more committed to her goals.

Serving as another unique motivational regulation strategy, sharing and learning, are closely tied to the inherent properties of OSM. Sharing is typically seen as altruistic but also benefits learners. Recording and sharing learning their progress serves as a “check-in” mechanism, creating a sense of accountability and promoting commitment to the learning path. In addition, sharing experiences is also a beneficial self-reflection method. As Julian put it, “some sharers hold the idea of learning by teaching.” Those who are willing to share learning experiences and tips are assumed putting a lot of effort into preparing their content before sharing it. By constantly refining their work, they actually improve interpreting skills over time. “It’s kind of like the whole ‘fake it until you make it’ thing, which I think can be pretty effective in self-learning.” Due to the seamless feedback mechanisms of OSM, sharing acts as a regulatory strategy of SRIL. Nearly half of learners boost motivation through sharing, feeling observed and praised by potential audiences, creating a spotlight effect not achievable offline.

Notably, OSM also serves as a virtual “learning venue,” as some learners actively utilize and reconstruct its functions to transform it into a space for practice. Joy gave her example of building a personalized learning environment by Weibo:

When I browse Weibo every day, if I come across practice resources mixed in with entertaining content, I’ll repost and tag them as “interpreting” or “listening” for practice the next day. I don’t want to bother others, so I make them visible only to myself.

The entertaining characteristic of OSM also serves as a great motivation booster. Interpreting practice, given its inherent complexity, often induces greater frustration among learners compared to general language acquisition. Many learners reported how they were demotivated when encountered negative feedback and frustrations during offline learning. The leisure-oriented nature of OSM effectively mitigates the perception of difficulty. As a result, interpreting practice appears less arduous and more like a leisurely engagement. As Wendy, a 23 years old learner shared:

When I was preparing for the grad school entrance exam, I would constantly glance at experience posts (of interpreting) and check out other news. Since I love browsing, doing this made me feel quite productive while entertaining.

Derived from its entertaining nature, OSM also provides emotional support, thus boosting learners’ motivation. Most interpreting learners do not strictly delineate between learning and life on social media, similar to when this generation first encountered self-media. OSM is dubbed a “secret garden.” While browsing others’ content, learners also share their own interpreting-centered lives, venting emotions, and seeking comfort and assistance. After 2 years as an MTI student, Marine is now a translator and interpreter for a Central Ministry, she expressed:

What I’m sharing these days is mostly about the stuff I run into while interpreting, like the hiccups on escort interpreting, the fun moments, food adventures. Just jotting down things that mean a lot to me to capture the unique moments in my interpreting journey.

In line with Marine’s sharing, encountering diverse individuals, their stories, expertise, and tangible assistance can also have a similar “uplifting effect.” OSM content supports learners emotionally through self-efficacy, affinity, empathy, reassurance, and a sense of community. Joy told me:

I get a real sense that they love what they do. You know, after reading it, I’m just really happy for them. It’s like they’re living their dream, and it’s so inspiring and impressive. It just gives me a really good vibe.

Nevertheless, the psychological impact of OSM is bidirectional. OSM can also overwhelm learners with others’ lives and opinions. Sometimes, the community can become an imagined arena, demotivating learners with peer pressure. Ada, a 23-year-old learner, claimed:

Social media content really influences my emotion. It’s like we’re all learning the same stuff, but why somehow others seem to soak it up better?

Many share the same feeling with Ada, as about 80% (*n* = 23) of participants mentioned the anxiety exerted by OSM. Among these negative emotions, anxiety is the most prevalent, followed by feelings of awkwardness, self-doubt, and boredom.

Despite negative feelings, misinformation on OSM may also potentially hinder learners’ motivational development. Unverified resources on OSM may lead to misconceptions, especially those generated by inexperienced students. “Spending money on online classes taught by student interpreters is unreasonable, despite any initial promise they may show, as their skills are not yet fully developed,” warned Miya. A 25-years-old student interpreter, Angela, echoed:

A lot of influencers selectively share online. They might not spill everything, just the bits they’re really satisfied with. So, it ends up looking like their whole world is just perfect and everything they do is a breeze.

In this case, it is imperative for novice learners to cultivate the ability to discern credible information from that which is misleading.

### 4.3 To be or not to be: self-reflection, perseverance and exit behavior

In the self-reflection phase, learners naturally progress to self-reaction, wherein they assess whether their objectives have been met and determine subsequent actions based on the outcome. For the majority of learners, the capabilities of self-evaluation and self-judgment rely on copious feedback. It is safe to say that interpreting learning, as skill acquisition, revolves around accumulation of declarative domain knowledge and sharpening interpreting skills. Practice, experiential learning, and effective feedback drive this transformation. As Miya stressed, “We need those spicy comments in class; they really push me to practice more on OSM. One cannot practice in vacuum.”

Intriguingly, the interactive potential of OSM does not appear to be fully realized in the context of interpreting learning. Many participants argue that the main disadvantage of OSM is the absence of interaction and effective feedback. Only 10.3% (*n* = 3) of participants claim to have received feedback from OSM. This ineffective feedback loop can worsen motivational issues caused by misinformation. Developing discernment requires interactive learning. Without diverse interpreting examples for comparison and reflection, learners struggle to distinguish good from bad. Julian provided an example illustrating a common pitfall for beginners:

I know some really rely on translations of audio transcription on OSM. But these often end up being just word-for-word machine translations. If you blindly follow that without critical questioning, you might end up practicing in a way that doesn’t really help you improve, or worse, sends you off in the wrong direction.

However, OSM can serve well as a complementary tool. Upon receiving feedback from their instructors, learners actively consult OSM for content addressing similar issues, their respective solutions, and further reflections. This enables the process of causal attribution, enhancing the learners’ motivation.

Once positive feedback is received, learners often undergo introspection and set new benchmarks for their journey toward professionalism. This phenomenon is particularly evident among MTI students, as highlighted in Angela’s insights:

In class, we’ve talked a lot about exams and exercises. Honestly, I’ve learned pretty much of those stuffs at the grad stage, but haven’t landed a job yet. You could say I am in an interface, so I really need someone who’s been through it to show us how to gear up for what’s next.

The goal-orientated motivation for SRIL demonstrates pronounced stages of change. For those who renewed their goal to a higher level, they are motivated to test the effectiveness of exercises by real-world practice. Chris provided her perception, “In simultaneous interpreting, sometimes you gotta let go of some details to stick to the main point. We talked about it in class too. But seeing professionals who’ve actually done it, sharing their tips. That just feels way more helpful.” Marine also extended this point with an example in her work:

I once found a new machine that I’ve never encountered in my booth. I quickly looked it up on Little Red Book, and found out how it operates. Using machines for in-class practice isn’t the same as the real deal. If your machine breaks during exercise, no big deal, because there’s no real audience so you’re not responsible for what you delivered. But in a real task, the equipment matters. Not knowing how to use even one button causes super anxiety.

Now as novice practitioners, learners adopt a knowledge system in interpreting that is less systematic but more flexible, guided by completing their work. Despite providing professional insights, OSM can interestingly become a job broker seeking in the employment-oriented stage. Smile, a 26 years old learner approaching her graduation, told me, “Right now, I’m mostly interested in job and internship info on social media, especially anything related to interpreting.” In this stage, OSM acts as a job assistant, boosting learners’ motivation for transitioning to professional interpreters.

Lastly, a significant pattern observed is the “exit behavior,” which represents a final stage where learners become highly demotivated and discontinue their practice. Among the participants in our study, predominantly current or former students of MTI programs, only a minority (20.7%) aspired to become professional interpreters. Isabella, a 24-year-old interpreting student, articulated her perspective:

Back in my undergrad days, I really wanted to be an interpreter and was keen on going to ABC school (a well-known interpreting training institution in China). But as I moved on, I started to rethink what I want to do in the future. One thing’s for sure, though – I won’t be pursuing interpreting as my main job.

The volatility of the interpreting job market might contribute to this phenomenon. Julian remarked, “The job market for young interpreters is challenging, making it difficult to secure suitable employment.” In this way, OSM somewhat demystifies the allure of interpreting, reducing its “symbolic capital” and “halo effect.” Therefore, demotivated learners often reassess their initial aspirations and explore alternative career paths.

In Self-Reflection stage of SRIL, OSM reveals that evolving job market pressures and alternative career paths lead learners away from their initial aspirations. However, interpreting learning still provide valuable skills and versatile competencies for career paths beyond traditional roles.

## 5 Discussion

This study investigates the role of OSM in SRIL, with a specific focus on interpreting learners’ motivation, its regulation and transformation. Situating our findings within the existing literature, we integrated some of the most interesting themes emerged from the interviews, particularly highlighting their relevance in the SRIL process.

Overall, we have identified the influence of OSM as a motivator (or deterrent), OSM as a bridge between learners’ motivations and actions, and OSM as a motivation regulator. These three themes are interconnected sequentially, as motivation needs to be translated into action, whereas the motivational regulation process develops, modulates and terminates actions. Concerning the two cognitive dimensions of motivation, namely as an end-state product and an on-going process (Wolters, 2003; Winne and Marx, 1989), the findings of this study intriguingly show that OSM plays a significant role in both, encompassing sociological, pedagogical, and psychological aspects. Additionally, the findings also indicate that OSM can impact motivation both positively and negatively, potentially preventing motivation from translating into action.

### 5.1 OSM as a (de)motivator in SRL

Our findings indicate that OSM can be a significant motivator for interpreting learners. Unlike general language learning, interpreting has a distinct career-oriented focus. OSM leverages the “symbolic capital” (Bourdieu, 1977) of interpreting, enhancing the overall visibility of interpreting as an industry, field, and career, thereby further motivating learners to pursue this profession.

Increased visibility of interpreters, driven by OSM dynamics, emerged as one significant theme. This is due to the fact that user-generated content on OSM has overridden conventional by mass media content, fostering a nuanced interplay between pseudo-environments and reality. It is worth noting that the transformation of interpreters’ image in China underwent several stages, as for much of the last century, interpreters were predominantly perceived by the Chinese public as in-house professionals affiliated with government units. There figures always appeared on television screens beside state leaders during political and diplomatic events—thus earning the title “translator officer” (翻译官, *Fanyiguan*), a term sometimes carrying negative connotations. On the contrary, today’s interpreters, as active influencers on OSM, discard their “official” masks and reveal the different aspects of their lives. These attributes form a certain degree of “illusio” (Bourdieu, 1977) of the interpreting profession in would-be interpreting learners’ motivation.

In line with such an “illusio,” Kotze (2024) conducts a pioneering study on how OSM discursively constructs the image and conceptualization of “translators,” who previously emerged as “shadowy figures” and “invisible servants” but are now empowered by OSM to represent their personal identities online as a “plot device” (2024, p. 18). Our findings suggest that interpreters exactly utilize OSM are their “plot device.” Interpreters utilize OSM to “break out of a niche” (出圈, *Chuquan*) to exhibit their efforts and expertise to a wider audience. Interestingly, aspiring learners find successful interpreting learners (majorly student interpreters) even more relevant and popular than professionals. By sharing their own learning experiences, outlining a replicable path to success, and visualizing career prospects, content producers on OSM add to the symbolic capital to interpreting as a career possibility, thus attracting more new comers. This visibility functions as a significant external motivator, and in turn cultivates learner’s intrinsic motivation. As a result, interpreting is increasingly perceived as a “social ladder” (Lung, 2005, p. 230) and seemingly offers highly paid and respectable employment opportunities.

Through a sociological lens, OSM potentially blurs the boundary between professionals and amateurs, by offering more equal access to aspiring interpreting learners. However, reality not always work in a positive way. The dynamics of OSM prompt us to consider whether the increased influx of interpreting learners will lower the barrier to entry into the interpreting market, further integrating interpreting into the broader language service industry rather than maintaining its independence and autonomy. Furthermore, self-initiated learning on OSM could potentially intervene aptitude testing, as it has not been tested whether OSM genuinely motivates and shapes interpreters, or merely creates a “camouflage effect.” To individuals, OSM can also dissolve their motivation, as the lagging regulatory system may breed misinformation, which may cultivate false habits to interpreting learners. Lastly, after gaining an understanding of the real market, some individuals may assess their abilities and decide to leave the industry.

### 5.2 OSM as a bridge between learners’ motivations and actions

Increased OSM visibility of interpreters and interpreting learning may inspire reconceptualization of interpreting pedagogy. OSM provides learning and, while not directly tied to motivation or its regulation, plays a significant bridging role and reshapes current interpreting pedagogy.

In OSM and educational settings, affordance can be defined as cues for action or action potentials evoked by multiple technologies in the learning environment, dynamically changing based on students’ learning goals, tasks, and interactions with other students and course facilitators (Dabbagh and Kitsantas, 2012). Once learners set clear goals, they start to evaluate the gap between their current conditions and what is needed to achieve them. One frequently mentioned drawback is subpar in-class teaching, which turns out to be the foremost obstacle. This concern is substantiated by findings from the most recent survey conducted by the Translators Association of China (TAC, 2018), which revealed that, despite the relatively low threshold of requiring fewer than ten real-world interpreting assignments for qualified instructors in accredited MTI programs, 73.7% of faculty members in the surveyed institutions failed to meet this criterion. Such findings underscore persistent concerns regarding the overall quality and pedagogical effectiveness of translation and interpreting (T&I) education. By providing accessible “learning affordance,” OSM rises to the occasion as a third-party strength, pushing students from initial motivation to sustained engagement.

Compared to general language learning, interpreting features more complex and personalized skills, necessitating diverse and tailored methods that OSM’s pedagogical affordances can effectively provide. For instance, OSM provides a Shared Repertoire (Valtchuk and Class, 2021) of SRIL, which constitutes the basis of learning affordance. This repertoire enhances learners’ metacognitive strategies by offering a broad range of guidance through “experience posts” of ideal planning. It also encompasses training materials, methods, and job-related information to enhance learners’ mastery. OSM also facilitates learners’ social behavioral strategies by indicating whom they can reach out to for assistance, particularly “significant others” (Horváth and Kálmán, 2020), which serves as a crucial factor for both motivation and regulatory processes. For example, second hand experience provided by practicing interpreters appears more persuasive than classroom lectures, which echoes the suggestion proposed by Chang and Wu (2017) that “interpreting students might subscribe to interpreters’ blogs and befriend interpreters online.” Real-world assignments or interpreting-related opportunities emerging from OSM serve as stronger motivators for learners, as they perceive their time and effort to be meaningfully invested and directly linked to tangible outcomes.

Online social media also functions as an interface between formal and informal learning environments (Dabbagh and Kitsantas, 2012), which is in line with Wolters’s (2003) proposal that environment structuring is a significant motivational regulation strategy. On OSM, learners’ practice environments are poised to be highly personalized, but besides self-practice, joining learning groups on OSM also serves as an effective regulatory strategy. For instance, WeChat group chats function as hubs for the circulation of study-related content. Compared to short-term offline interactions, a multi-person environment on OSM offers more effective supervision and regulation due to its capacity for immediate feedback, as peer interaction cultivates an environment of support and encouragement, which can strengthen learners’ motivation and confidence as they set learning goals and plan their interpreting practice—it echos the findings on Facebook group chat of Valtchuk and Class (2021) that OSM also serves as a learning venue on cyberspace, assembling learners’ personal learning environments. In this sense, OSM serves not only as a resource repository but also as a dynamic platform that stimulates engagement and planning in the early stages of SRL, effectively promoting learners’ motivation and readiness for interpreting tasks. However, by utilizing multiple functions of OSM, individuals tend to create the most authentic learning environment (González-Davies and Enríquez-Raído, 2016; Chang and Wu, 2017) for themselves, through which they proactively regulate their motivation.

More intriguingly, OSM introduces two relatively unconventional types of social behavioral affordances, both of which emphasize external influence. The first involves paid tutorials, which operate as a form of externally imposed self-consequencing to regulate motivation. The second, “sharing as learning,” is driven by intrinsic motivation and acts as a powerful impetus for sustained learning, while learners’ will of sharing is largely shaped by external user feedback.

### 5.3 OSM as a motivation regulator

Our findings also suggest that OSM serves as a crucial regulator of motivation, particularly in emotional control. The demanding nature of interpreting induces significant stress and anxiety both in practice and in learning; thus, during the process of motivational regulation, the role of OSM in managing emotions is particularly important. To persevere in SRIL, learners strive to regulate and maintain their initial motivations, a process largely influenced by psychological factors.

Motivational regulation strategies embody various actions or tactics through which individuals can consciously control their affect and emotion (such as stronger self-efficacy and reduced helplessness) to initiate, maintain, or supplement their willingness or effort when completing a learning activity (Zimmerman and Schunk, 2008; Teng and Zhang, 2017). Whether they actively seek connections or are passively connected, learners use OSM to maintain a stable mindset. At the initial stage of the learning process, learners often—whether consciously or unconsciously—seek out role models as “significant others” (Horváth and Kálmán, 2020), to serve as sources of inspiration and aspiration. According to Tajfel and Turner’s (2000) social identity theory, observing the success of peers, particularly those who share similar backgrounds, can significantly enhance learners’ motivation by reinforcing a sense of attainable achievement and social belonging. After that, as they become situated within a network of diverse individuals, they also constantly draw emotional support from those in similar circumstances and receive warm encouragement, again echoing the concept of “significant others.”

Moreover, the entertaining nature of OSM helps learners discover the multifaceted and intriguing aspects of interpreting, thereby better regulating their intrinsic motivation. Attribution control on OSM, acting as a feedback loop from internal to external attributions, enables learners to timely identify problems, releasing them from helplessness, self-doubt, and anxiety. Lastly, we extend the existing understanding of altruism in SRIL, particularly as it pertains to sharing behaviors, demonstrating that it meets the needs of certain learners. This finding contradicts Horváth and Kálmán (2020) finding that altruism is a drawback. In fact, some active users adopt a “sharing as learning” strategy to enhance their self-efficacy. Regardless of its authenticity, the persona of a competent interpreter (or interpreting learner) that learners cultivate on OSM motivates them to strive harder toward achieving their goals.

Learners proactively use all of the aforementioned functions of OSM to reduce their negative feelings, such as anxiety, during SRIL. OSM creates a personal learning space that offers privacy from teachers (Valtchuk and Class, 2021), alleviating potential shame associated with offline teaching and reducing reluctance during interpreting practice. Once interpreting is perceived as manageable and achievable, learners can better regulate their motivation, embarking on their practice journey with more confidence.

However, OSM also has two major psychological drawbacks. One points to growing pessimism regarding the future of the language services industry—a sentiment largely fueled by the rapid advancement of large language models (LLMs) such as ChatGPT, from which the interpreting profession is not immune. Regardless of its empirical validity, this perception is further amplified by content disseminators on OSM. The second is the anxiety induced by peer comparison. The prevalence of highly curated, idealized portrayals of professional success on these platforms may inadvertently undermine learners’ confidence, particularly among those who are more emotionally vulnerable.

## 6 Conclusion and future work

Based on the Cyclical Model of SRL (Zimmerman, 2000), this study provides substantial and detailed qualitative data to explore the motivation of Chinese interpreting learners to voluntarily engage in SRIL on OSM, and the formation, evolution, regulation and dissolution of their motivations. Overall, the findings highlight the dynamics of OSM as a motivator, as a bridge between learners’ motivation and its regulation, and as a regulator of motivation, corresponding to sociological, pedagogical, and psychological aspects. The findings also reveal that for the first time that these factors on OSM not only positively influence motivation but may also, in reality, hinder motivation from further action.

This study contributes to the ongoing discussion on SRL by extending Zimmerman’s (2000) Cyclical Model to the context of OSM-mediated learning. It highlights the dual role of OSM as both a motivator and a deterrent, suggesting a nuanced understanding of motivational regulation that includes the influence of digital platforms. While motivation as a construct has been widely studied in classroom settings, this research focuses on how learners self-regulate their motivation in less formal, digitally-mediated environments. These findings are relevant to educators and researchers globally, as the dynamics of motivation and SRL apply across educational contexts, from formal institutional settings to informal online communities, including diverse global contexts where access to formal training is unevenly distributed.

Several practical implications for optimizing the use of OSM can be drawn that maximizes its benefits while addressing its challenges. First, integrating OSM into formal interpreting education can enhance motivation by providing diverse, real-time, and authentic learning experiences. In this sense, it also enables educators to identify exemplary and replicable learning trajectories from OSM content, which can be used to construct relatable role models—by virtually “befriending” these figures, students’ motivation can be further boosted. Moreover, OSM can also democratize access to interpreting resources, especially for learners in regions where formal education resources are limited, allowing educators to incorporate selected high-quality materials and relevant approaches from OSM content to enrich students’ understanding of real-time interpreting. Finally, encouraging students to share their learning on OSM can cultivate a habit of learning by sharing, fostering a more collaborative and supportive online environment. During this process, educators must also develop students’ critical thinking skills to navigate the potential drawbacks, such as misinformation and distraction, often associated with OSM. In these ways, leveraging the strengths of OSM both in and beyond the classroom can naturally bolster and maintain motivation in interpreting practice—After all, integrating OSM into pedagogy fosters lifelong learning skills, supporting students beyond formal education environments and preparing them for continuous professional development.

The study also has limitations. While the qualitative approach provides depth and context, it also faces subjectivity and the inability to provide statistically generalizable findings. The sample’s representativeness requires enhancement, as it majorly consists of graduate students from leading universities. Future research should explore specific aspects of the OSM ecosystem, such as algorithms and content producers, and investigate how OSM-driven motivation correlates with learning behaviors, performance, and professional success. A mixed-methods approach, incorporating quantitative data, would offer a more comprehensive understanding of these relationships and complement the qualitative insights of this study.

## Data Availability

The datasets presented in this article are not readily available because the dataset analysed in this study will be provided by the authors upon reasonable request. Requests to access the datasets should be directed to SY, sunshineyang0168@163.com.

## Footnotes

1 As one of the major Chinese over-the-top video-sharing sites, Bilibili can be regarded as “Chinese YouTube,” known for its focus on animation, gaming, and entertainment. The primary form of user-generated content on Bilibili is self-filmed videos.

2 European Commission. “Speech Repository.” Accessed July 29, 2024. https://speech-repository.webcloud.ec.europa.eu/.

3 Sina Weibo is a twitter-like social media platform in China, known for its microblogging format where users usually share short posts and images, and is characterized by a diverse user base that includes celebrities, influencers, and individuals.

4 Xiaohongshu is the Chinese counterpart of Instagram. It has become the most popular lifestyle platform in recent years in based on user-generated short post, images and video.

5 WeChat is a leading social media platform in China similar to WhatsApp featuring its Official Accounts, which primarily sharing text-based content, allow businesses, media, and individuals to publish articles, updates, and multimedia.

6 Bao Gaogao and his spouse, Wen Niuniu, are distinguished interpreters and vloggers with a substantial follower base, actively engaging on several prominent Online Social Media platforms in China, including Xiaohongshu, Sina Weibo (Sina Microblog), and Bilibili.

7 “Summer Camp” constitutes an integral component of the early admission initiative for graduate students within Chinese higher education institutions.

## Appendix

**APPENDIX 1:** Interview protocol for participants.

Demographic information
How old are you?
What is your gender?
In which city and school are you working for your undergraduate degree in?
In which city and school are you working for your postgraduate degree in?
What is your undergraduate major?
What is your postgraduate major?
How long have you learned interpreting?
Have you taken any interpreting related exams or real-world interpreting assignment?
**The Main Interview Questions**
•**Describe your motivation before learning interpreting on OSM**
When was the first time you heard about interpreting, and your impression of it?
Why do you choose interpreting as your graduate major?
What led you to interpreting-related content on OSM?
What interpreting-related accounts do you follow and why?
•**Describe your motivation during interpreting learning on OSM**
In what occasions would you utilize OSM to assist your interpreting learning?
Tell me a story about OSM’s role in your interpreting learning process.
What type of interpreting-related OSM content do you pay the most attention?
Will OSM make you more or less want to practice interpreting? Could you specify?
How do you perceive OSM’s role compared to offline teaching?
•**Describe your motivation after interpreting learning on OSM.**
In your opinion, is OSM helpful for your real-world interpreting work and employment or not?
Do you (intend to) engage in interpreting related work? Why or why not?
In your opinion, does OSM motivate you to be a professional interpreter? Why or why not?
Tell me a story about how OSM content influences your choice of career path.

**APPENDIX 2:** Example of initial coding process.

**I (interviewer):** Honestly, this is something new to me. Why do you think it happens?	
**P (participant):** I think it’s more of an illusion created by new media; it’s not entirely accurate.	^1^ILLUSION
**I:** You mentioned that you search for resources online, but they might not be high-quality. So, do you agree that to secure a good interpreting gig, it’s still best to rely on personal connections?	
**P:** Yes, based on my experience and feedback from friends, many interpreting job postings online are of low quality and poorly paid. The requirements are often minimal, meaning these jobs aren’t suited for top-tier interpreters. Additionally, many of these postings are second-, third-, or even fourth-hand information.	^2^ LOW QUALITY INFORMATION ^3^ LOW PAYMENT ^4^ LOW REQUIREMENT ^5^ MULTIPLE LAYERS OF OUTSOURCING
**I:** It’s like multiple layers of outsourcing, and by the time it reaches you, there’s hardly any money left.	
**P:** Exactly. Not only is the pay low, but the information is often outdated. It might have been shared in another group and then forwarded here, making it less reliable. Constantly monitoring these groups can also be a significant source of anxiety.	^6^ LOW PAYMENT ^7^ OUTDATED INFORMATION ^8^ SHARING ACROSS MULTIPLE GROUPS ^9^ LACK OF RELIABILITY ^10^ SOURCE OF ANXIETY
**I:** Speaking of anxiety, I’m curious—do you feel anxious when you see others on social media constantly posting about their interpreting gigs?	
**P:** Absolutely. That’s why I eventually stopped looking at those posts.	^11^ OSM EXIT TO AVOID ANXIETY

**APPENDIX 3:** Example of coding process of forethought stage.

Sub-theme	Initial codes	Number of participants	Number of citations	Example quote
Interpreters’ image	Independence of Life Language proficiency High payment **Flexible schedules** High social status Frequent international travel	5	7	I first heard about the word “interpreting” when my sister helping me select a college application. She told me, “You should study interpreting—it pays well— according to short videos.” (P23)
Subpar in-class teaching	Unsatisfying undergraduate interpreting courses Incompetent teachers Teacher-student perception gap Lack of training space Outdated textbooks	25	31	I did my undergrad in English at an agricultural university. To be honest, it was pretty crappy. I didn’t really learn much from classes. (P3)
Role model	Prediction of personal success Uplifting Effect The increased attainability of interpreting pursuits	20	22	Maybe seeing me pass made others think, “if she can do it, maybe I can too.” (P25)
Goal setting	Industry’s requirement for entry Professional or academic qualifications CATTI PGEE	29	34	Enrollment in an institutionalized program signifies the beginning of my interpreting journey. (P18)
“Experience post” guidance	Exam preparation, Time management strategies Advice on program selection Practice techniques.	28	51	I chose a day to dig up all the posts I could find on social media, putting together everything they shared, like what the exam was like, what was on it, and even the sharers’ academic backgrounds. (P23)
